# Earlier Onset of δ-Retrovirus-Induced Leukemia after Splenectomy

**DOI:** 10.1371/journal.pone.0006943

**Published:** 2009-09-14

**Authors:** Arnaud Florins, Michal Reichert, Becca Asquith, Amel-Baya Bouzar, Geneviève Jean, Carole François, Agnieszka Jasik, Arsène Burny, Richard Kettmann, Luc Willems

**Affiliations:** 1 Cellular and Molecular Biology, Gembloux Agricultural University (FUSAGX), Gembloux, Belgium; 2 National Veterinary Research Institute, Pulawy, Poland; 3 Department of Immunology, Imperial College, London, United Kingdom; 4 Zootechny Unit, Gembloux Agricultural University (FUSAGX), Gembloux, Belgium; 5 Interdisciplinary Cluster for Applied Genoproteomics (GIGA), University of Liège (ULg), Liège, Belgium; University of Minnesota, United States of America

## Abstract

Infection by δ-retroviruses such as human T-lymphotropic virus type 1 (HTLV-1) and bovine leukemia virus (BLV) is mostly asymptomatic. Indeed, only a minority (<5%) of δ-retrovirus infected hosts will develop either lymphoproliferative or neurodegenerative diseases after long latency periods. In fact, the host immune response is believed to tightly control viral replication but this assumption has not been definitely proven *in vivo*. Here, we provide direct experimental evidence demonstrating that integrity of the spleen is required to control pathogenesis. In the BLV model, we show that asplenia decreases efficiency of the immune response and induces an imbalance in cell dynamics resulting in accelerated onset of leukemia. These observations enlighten a potential threat in splenectomized HTLV-1 carriers and justify a regular preventive evaluation.

## Introduction

Surgical removal of the spleen is performed for a number of reasons, including trauma and different pathologic conditions such as hematological disorders, hypersplenism or splenomegaly. Although the risk of overwhelming postsplenectomy infections by encapsulated bacteria has been clearly documented [1], [2], the question whether asplenia plays a role in cancer development have yielded conflicting results [3]–[9]. On one hand, no increase in cancer risk was detected among patients having experienced a splenectomy for trauma [9]. On the other hand, clinical evidence indicates that splenectomy increases the risk of secondary breast cancer and acute nonlymphocytic leukemia in patients treated for Hodgkin's disease [10]–[12]. Accelerated leukemia/lymphoma progression has also been reported after splenectomy in NK cell leukemia and in large granular lymphocyte leukemia [13], [14].

Among 20 million HTLV-1 infected subjects worldwide, a proportion will undergo splenectomy for traumatic or diagnostic purposes. Since complications of asplenia in HTLV-1 carriers are presently unknown and cannot be experimentally tested, we evaluated the risk associated with splenectomy in an animal model: bovine leukemia virus (BLV)-infected sheep. HTLV-1 and BLV are two related δ-retroviruses infecting CD4+ in humans and B lymphocytes in ruminants, respectively [15]. Both viruses share common epitopes in the *gag*-encoded capsid antigens further supporting a close evolutionary relationship between the two viruses. Genomic organization and gene functions are also remarkably similar: in addition to the structural *gag*, *pol* and *env* genes required for the synthesis of the viral particle, both viral genomes contain a particular region pertaining to the δ-retrovirus genus and coding for regulatory proteins. Common features of HTLV-1 and BLV pathogeneses include horizontal transmission, cell-associated infection, random integration into the host genome, dual routes of viral replication (i.e. mitotic and infectious) and accelerated cell turnover.

In view of the similarities between the two viruses in terms of pathogenic mechanisms, we think that improved understanding in the BLV model may be informative for HTLV-1. In this context, we addressed the role of asplenia in BLV-infected sheep.

## Methods

### Ethics statement

Handling of sheep and experimental procedures were approved by the ethic committee (local ethic committee of Gembloux Agricultural University; C.L.E.) and were conducted in accordance with institutional and national guidelines for animal care and use.

### Experimental animals, splenectomy and sample collection

50 sheep were maintained under restricted conditions (L2) either at the Veterinary and Agrochemical Research Center (Machelen, Belgium), the Study Center of Animal Productions (Gembloux, Belgium) or the National Veterinary Research Institute (Pulawy, Poland). 40 sheep were experimentally infected with the wild type BLV strain 344 [16]. Ten BLV-infected sheep and 3 controls were splenectomized at 4.6±3.2 months post-infection. For splenectomy, sheep were anesthetized by intravenous injection of approximately 10 ml of Nembutal (50 mg/ml, Abott Laboratories), followed by a perfusion of 2 ml/hour during the surgery. The spleen was then removed after ligature of the splenic vein and artery of sheep. At regular intervals of time, blood samples were collected by jugular venipuncture, mixed with anticoagulant (either with 0.3% w/v of EDTA or 10 U/ml of heparin). Leukocyte and lymphocyte counts were determined using an automated cell counter (MS 4-5 vet Mellet Schloesing Laboratories). Peripheral blood mononuclear cells (PBMCs) were isolated by Percoll density gradient centrifugation (GE Healthcare) and washed twice with PBS/0.075% EDTA and at least three times with PBS alone to eliminate platelets. PBMCs were either cryopreserved in DMSO/fetal calf serum (10/90 v/v) or directly used for analysis.

### *In vivo* cell kinetics measurement

*In vivo* cell turnover parameters were estimated by intravenous injection of 5-bromo-2′-deoxyuridine (BrdU) and carboxyfluorescein diacetate succinimidyl ester (CFSE). BrdU incorporates into the DNA of cells undergoing proliferation mainly in lymphoid tissues whereas CFSE labels ≥98% of resting and proliferating peripheral blood mononuclear cells [17], [18]. PBMCs were prepared at regular intervals of time and labeled with a monoclonal antibody directed against immunoglobulin M (anti-IgMs, mouse IgG1) and with a rat anti-mouse IgG1 phycoerythrin (PE)-antibody (Becton Dickinson Immunocytometry Systems). For BrdU labeling, cells were fixed with PBS/4% paraformaldehyde, treated with FACS permeabilizing solution (Becton Dickinson Immunocytometry Systems), and stained with anti-BrdU FITC antibody in the presence of DNAse (Becton Dickinson Immunocytometry Systems). The percentages of BrdU+ and CFSE+ B cells were then determined by flow cytometry (FACS Aria, Becton Dickinson Immunocytometry Systems).

Proliferation and death rates were estimated by fitting mathematical models to BrdU and CFSE labeling data obtained by flow cytometry [17]–[19]. These models basically use two sets of data from the flow cytometry analyses: the proportion of labeled cells and the mean fluorescence intensities. Compilation of these two types of experimental data allows calculation of the proliferation and death rates, the latter parameter also including the disappearance of the cells out of the blood.

### Evaluation of immune response *ex vivo*

The principle of the technique is based on the antigenic stimulation of blood samples with BLV proteins expressed by fetal lamb kidney cells (FLK-BLV). Confluent FLK-BLV cultures (250 cells/µl) were lysed by 3 freeze-thaw cycles, dislocated by sonication, centrifuged for 10 minutes at 3000 g and stored in aliquots at −70°C. Heparinized blood was regularly collected and mixed with an equal volume of RPMI 1640 medium supplemented with 10% FCS, 2 mM L-glutamine, 100 U/ml of penicillin, 100 U/ml of streptomycin and 1 mM of sodium pyruvate. Blood and FLK-BLV precleared lysates were then mixed in a 4/1 proportion (v/v) and incubated in a 5% CO_2_-air atmosphere at 37°C during 5 days. After culture, brefeldin A (5 µg/ml) was added during 4 hours and red blood cells were lysed with 1X FACS Lysing Solution (Becton Dickinson Immunocytometry Systems) for 15 min at room temperature. Leukocytes were permeabilized in PBS-0.1% saponin, stained with monoclonal antibodies directed against CD8 (CACT80C, mouse IgG1) and perforin (DeltaG9, mouse IgG2A) and then labeled with rat anti-mouse IgG1-FITC and rat anti-mouse IgG2a+b-PE conjugates. Finally, the percentages of CD8+ perforin + cells were determined by flow cytometry.

### Determination of proviral loads and viral expression *in vivo*

Proviral loads were evaluated by real time PCR as previously described [20]. Quantification of BLV RNA expression was determined as follows: 500 ng of RNA were first reverse transcribed into cDNA using Euroscript reverse transcriptase (Eurogentec) and random nonamers. β-actin and Tax transcripts were then amplified by AmpliTaq Gold DNA polymerase (Applied Biosystems) on a Stepone apparatus (Applied Biosystems) using a pair of primers (400 nM of 5′-CAGCACAATGAAGATCAAGATCATC-3′/5′-CGGACTCATCGTACTCCTGCTT-3′ or 400 nM of 5′-GCCTTCAAATGCCTAAAGAACG-3′/5′-CGGGCAGGCATGTAGAGAGT-3′, respectively) and revealed with Black Hole Quencher splice junction probes (100 nM of 5′FAM-TCGCTGTCCACCTTCCAGCAGATGT-3′BHQ1 or 60 nM of 5′FAM-CAACAACACTTGCCATCTGATGATCG-3′BHQ1, respectively).

To determine the RNA copy numbers, β-actin and Tax amplicons were first cloned into plasmid pCR4-TOPO (Invitrogen) and transcribed *in vitro* (Megascript; Ambion). 10^2^ to 10^6^ RNA copies of each transcript were then reverse transcribed and amplified as described above. These standard curves allowed absolute quantification of Tax and β-actin copy numbers in samples.

## Results

**Figure 1:**
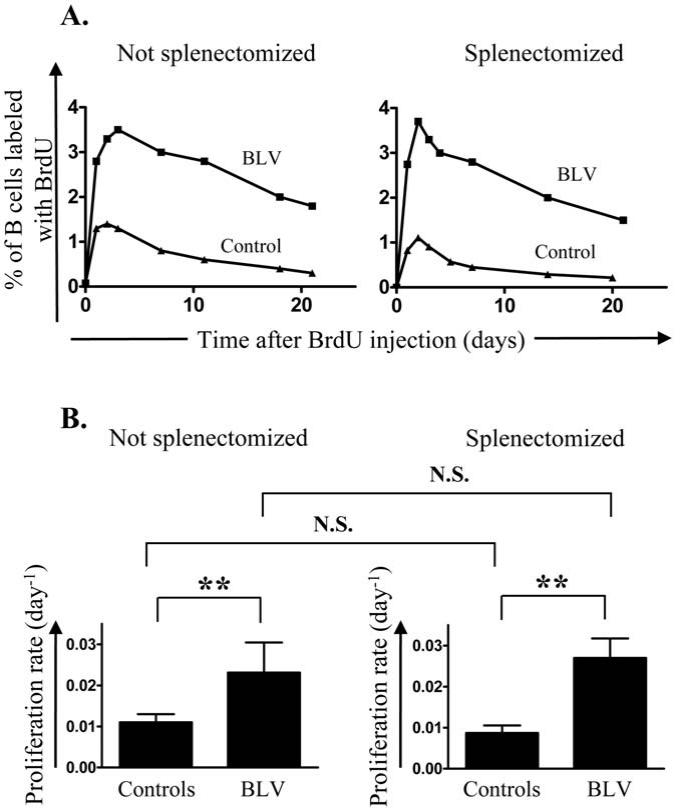
Evaluation of proliferation rates in vivo. BrdU was injected intravenously in BLV infected sheep and in non infected controls. At regular interval of time, the percentage of B lymphocytes having incorporated BrdU was determined by flow cytometry. A mathematical model was fitted to *in vivo* BrdU labeling data in B lymphocytes and proliferation rates were estimated. Not splenectomized (left panels) and splenectomized (right panels) animals were analyzed. (A) Percentage of B cells having incorporated BrdU in BLV-infected (▪) and control (▴) sheep. (B) Mean values (±SD) of proliferation rates. BrdU was injected in 13 infected sheep, 4 of them being splenectomized and 6 controls, 3 of them being splenectomized. Statistical relevance (n.s., not significant; p<0.01, highly statistically significant**) was calculated according to the unpaired two-tailed student t test with Welch's correction.

**Figure 2:**
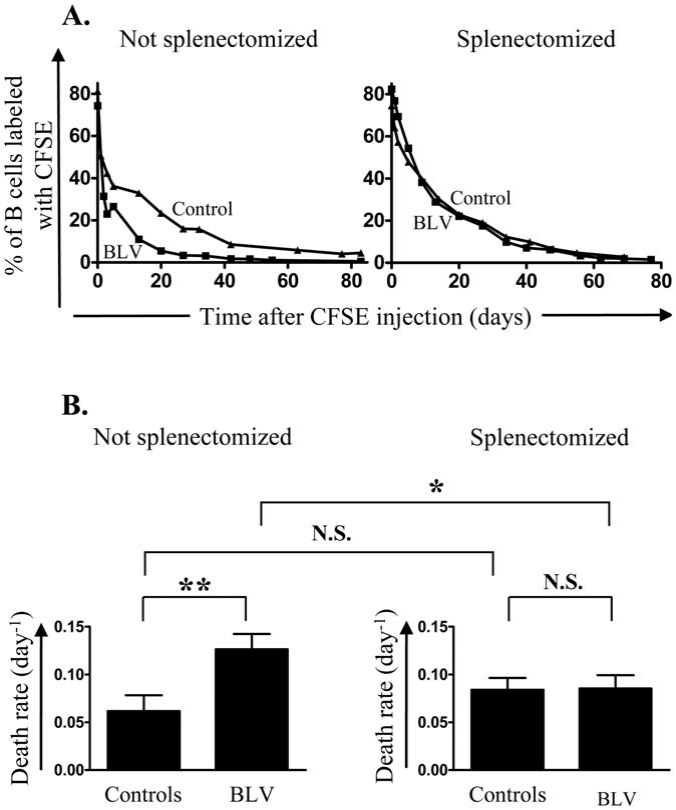
Evaluation of death rates in vivo. CFSE was injected intravenously in BLV infected sheep and in non infected controls. At regular interval of time, the percentage of B lymphocytes labeled with CFSE was determined by flow cytometry. A mathematical model was fitted to *in vivo* CFSE labeling data in B lymphocytes and death rates were estimated. Not splenectomized (left panels) and splenectomized (right panels) animals were analyzed. (A) Percentages of B cells labeled with CFSE in BLV-infected (▪) and control (▴) sheep. (B) Mean values (±SD) of death rates. CFSE was injected in 7)infected sheep, 3 of them being splenectomized and 7 controls, 3 of them being splenectomized. Statistical relevance (n.s., not significant; p<0.05, statistically significant*; p<0.01, highly statistically significant**) was calculated according to the unpaired two-tailed student t test with Welch's correction.

Cell proliferation and death can be quantified in BLV-infected sheep using CFSE and BrdU kinetic profiles. CFSE preferentially labels peripheral blood cells whereas BrdU is incorporated upon proliferation mainly in lymphoid tissues [17], [18], [20]. To evaluate the role of the spleen in B cell dynamics, BrdU and CFSE were injected intravenously before or after splenectomy in BLV–infected and control sheep. The proportion of cells labeled with BrdU (figure 1A) and CFSE (figure 2A) were then measured by flow cytometry at different times in non-operated (left panels) and asplenic sheep (right panels). The proportion of B cells having incorporated BrdU reached a maximum (3.5% at day 3 in a representative infected sheep ▪ and 1.4% at day 2 ▴ in a control; figure 1A, left panel) and then progressively decreased. Mathematical modeling of BrdU experimental data in a series of sheep showed that B cell proliferation was increased in BLV-infected animals (figure 1B, left panel; p<0.01). Upon splenectomy, this difference in B cell proliferation between BLV-infected and control sheep was conserved (figure 1B, right panel; p<0.01).

Next, the disappearance rate of peripheral blood B cells was quantified by CFSE kinetic profiles (figure 2A). Intravenous CFSE injection labeled more than 98% of peripheral blood B cells independently of BLV infection and splenectomy (data not shown). Modeling these CFSE data showed that, in eusplenic sheep, B cells died significantly more rapidly in BLV-infected than in control sheep (figure 2B, left panel; p<0.01) and that this difference in death rates disappeared upon splenectomy (Figure 2B, right panel; N.S.). Among BLV-infected sheep, splenectomy induced a significant decrease in cell death rates (Figure 2B, compare left and right panels, p<0.05).

**Figure 3:**
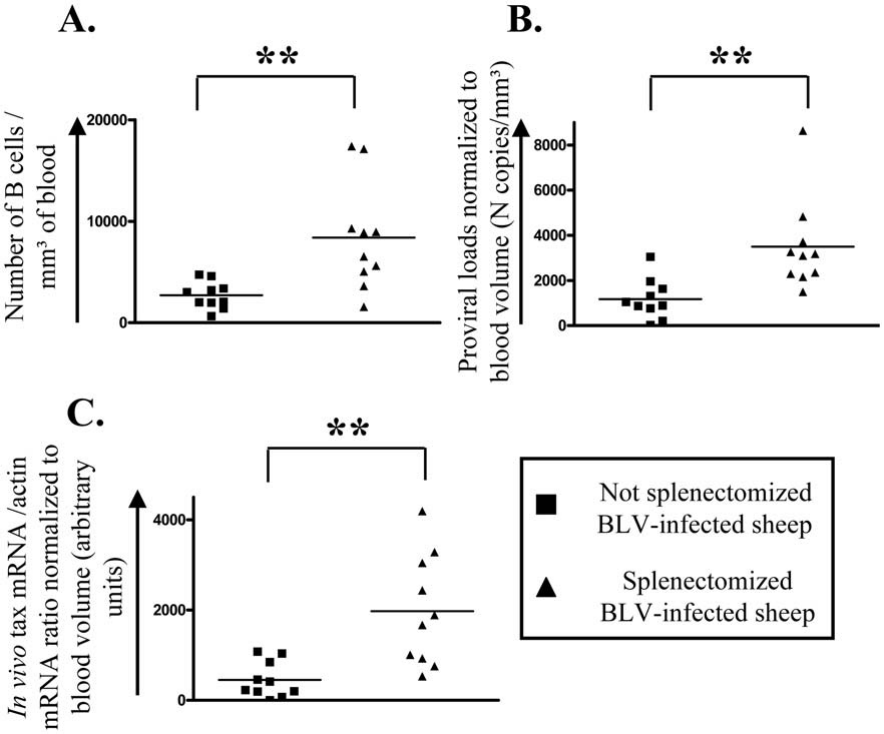
Influence of splenectomy on B cells counts, proviral loads and in vivo viral expression. 20 sheep were infected with BLV. A few months after seroconversion (average delay: 4.6 months), 10 of them were splenectomized. Blood was collected at regular intervals of time and different parameters were measured. Each value represents the average of 3 representative data in not splenectomized (▪) and splenectomized (▴) animals. (A) Numbers of B lymphocytes/mm^3^ of blood. (B) Proviral loads (in numbers of viral copies/mm^3^ of blood were determined by real time PCR and normalized to the blood volume. (C) Proportions between tax and actin transcripts (in arbitrary units) were measured by real time RT-PCR and normalized to the blood volume.

Fitting all kinetic parameters together (compare panels 1B and 2B) thus reveals an imbalance of the cell turnover in BLV-infected asplenic sheep. To correlate this modification in cell dynamics with viral replication, the proviral loads were compared in 20 BLV-infected sheep, 10 of them having been splenectomized. Consistently, splenectomy increased the numbers of B lymphocytes in the peripheral blood (Figure 3A; compare ▪ and ▴; p<0.01). Asplenic animals also had significantly higher DNA proviral loads and increased viral expression (Figure 3B and 3C; p<0.01).

**Figure 4:**
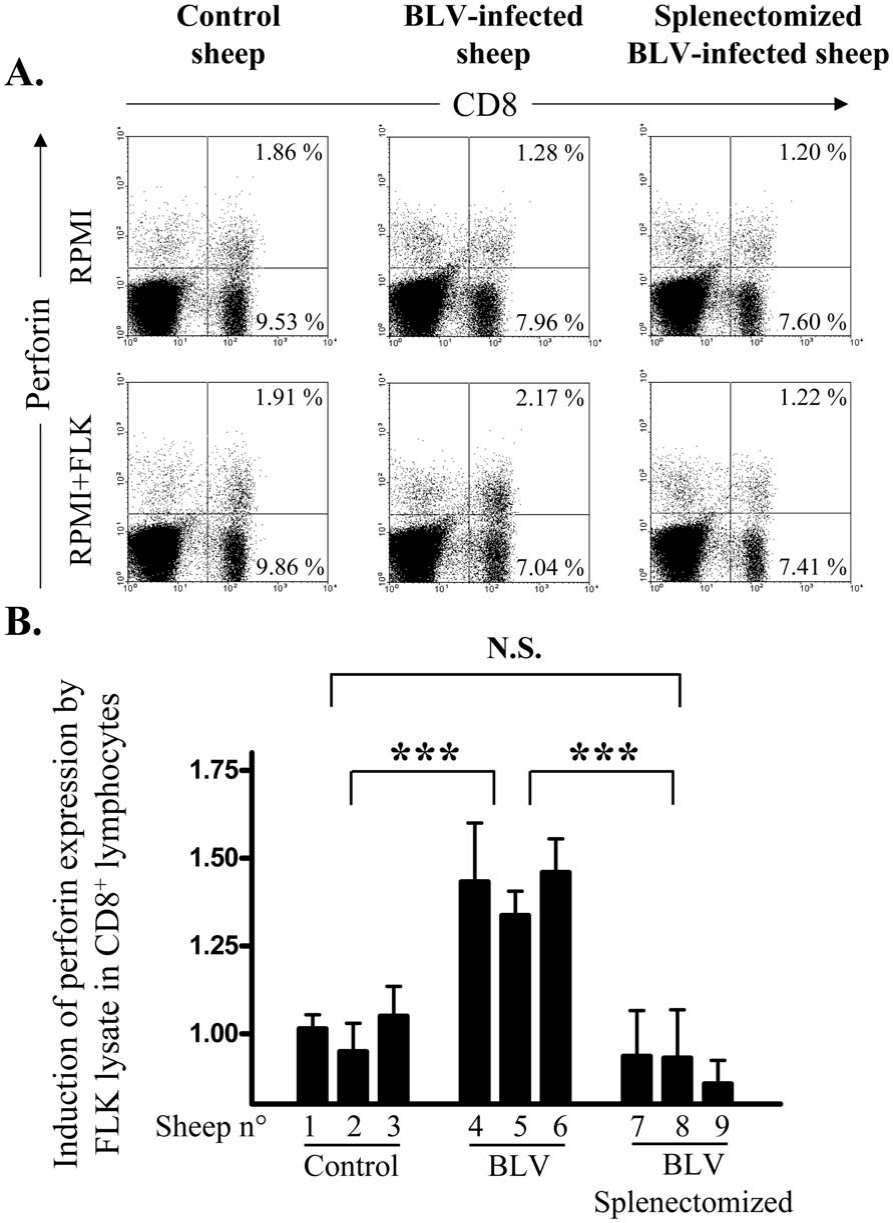
Induction of perforin expression in CD8 lymphocytes. Heparinized blood was incubated in RPMI medium in presence or absence of a lysate from FLK-BLV cells. After 5 days of culture, brefeldin A was added during 4 hours, leukocytes were permeabilized and CD8 and perforin expression were quantified by flow cytometry. (A) Representative dot plots of CD8 (x axis) and perforin (y axis) labeling of leukocytes from control, infected and splenectomized infected sheep, with and without antigenic stimulation. (B) Induction of perforin expression calculated as the ratio between the proportion of CD8 expressing perforin with and without antigenic stimulation. These values were measured 4 times (during a 3 months period) in control, BLV-infected and splenectomized BLV-infected sheep. Statistical relevance (N.S. not significant; *** very highly statistically significant p<0.001) was calculated according to the unpaired two-tailed student t test with Welch's correction.

The present knowledge of BLV replication postulates that the proviral load is tightly controlled by the host immune response and more particularly by cytotoxic T cells [21]. In this context, the increased proviral loads observed in splenectomized sheep may be due to a reduction of immune surveillance. Therefore, the ability of BLV antigens to stimulate CD8+ lymphocytes was compared in splenectomized and eusplenic sheep. The principle of the technique is based on stimulation of perforin expression by CD8+ T cells in presence of BLV proteins from a lysate of FLK-BLV cells. To closely mimic CTL response undergoing *in vivo*, the assay is performed in cultures of heparinized blood simply diluted twofold in RPMI medium. Representative flow cytometry analyses (figure 4A) show that the percentages of CD8+ cells expressing perforin upon antigen stimulation increase in BLV-infected sheep cultures (middle panels) but not in the control (left panels). Importantly, splenectomy abrogates perforin stimulation in CD8 lymphocytes from BLV-infected splenectomized sheep (Figure 4A, right panels). The same experiment repeated 4 times over a 3 months period in 9 sheep confirmed the lack of CD8+ activation after splenectomy (Figure 4B).

Together, these data strongly support a major role exerted by the spleen to control viral replication via a CD8+ mediated cell response. In contrast, no significant difference was observed in the virus-specific antibody titers (i.e. antibodies directed against the gp51 viral envelope protein, data not shown), further supporting a predominant effect of the cytotoxic response compared to the humoral immune surveillance.

**Figure 5:**
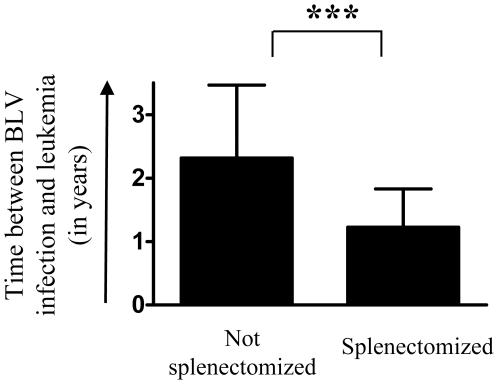
Development of leukemia in splenectomized animals. 10 BLV-infected sheep were splenectomized a few months after seroconversion (average delay: 4.6 months). Mean (±SD) latency periods (time between seroconversion and leukaemia; leukocytes counts >40 000/mm^3^) in non operated BLV-infected sheep (n = 29) and in splenectomized sheep (n = 9). Statistical relevance (p<0.001, very highly statistically significant ***) was calculated according to the unpaired two-tailed student t test with Welch's correction.

Failure of an efficient immune control of viral replication is consistent with an increase in proviral loads (Figure 3B) and a reduction in cell death rates *in vivo* (Figure 2B). It is also expected that favored viral replication induces a faster accumulation of infected B cells in asplenic animals (Figure 3A). Interestingly, amongst 10 splenectomized BLV-infected sheep, 9 developed acute leukemia (leukocyte counts >40,000/mm^3^) after unusually short latency periods (Figure 5). The mean delay between infection and onset of leukemia was only 1.23±0.60 years in asplenic sheep compared to 2.32±1.15 years in 29 non-splenectomized controls (p<0.001). These observations clearly assess a major role of the spleen in disease progression in BLV-infected sheep.

## Discussion

The most straightforward contribution of this paper is that splenectomy accelerates leukemogenesis induced by BLV in sheep. Assessment of *in vivo* cell kinetics (Figures 1 and 2) reveals a disequilibrium in cell death rates leading, in the absence of compensatory change in proliferation rates, to the progressive accumulation of infected cells in asplenic animals (Figure 3). Earlier onset of leukemia in splenectomized sheep also correlates with an impairment of a BLV-specific immune response (Figure 4). Our results therefore indicate that disease acuteness is linked to an impairment of cytotoxic response against BLV.

Although consistent with the data, this simplified model should be put into perspective. First, we cannot formally exclude that the humoral response does not control expansion of infected cells although antibodies directed against the gp51 viral envelope protein remain constant upon splenectomy. It remains indeed possible that the titers of other antiviral antibodies are modified in asplenic sheep. Second, the increased number of B lymphocytes observed after splenectomy is not specific to BLV infection. In fact, this surgery also increases the cell counts in non-infected sheep (data not shown) as observed in other species [22], [23]. Third, splenectomy does not appear to modify the capacity of the virus to be expressed *ex vivo* and *in vivo*. In fact, the relative proportions of BLV-expressing cells measured in short term cultures as well as the tax/β-actin mRNA ratio determined by real time RT-PCR are not significantly affected by splenectomy (data not shown). This observation indicates that the spleen integrity does not modulate viral expression in infected cells and suggests that the virus continuously attempts to replicate independently of the immune response. Fourth, susceptibility of infected B cells to undergo spontaneous apoptosis *ex vivo* is not modified by splenectomy (data not shown). Since CFSE kinetic profiles clearly demonstrate that the disappearance rates of infected B lymphocytes is reduced upon splenectomy (Figure 2), this data indicates that these cells have a similar intrinsic capacity to survive upon short term culture but are destroyed by the immune response *in vivo*. This elimination of infected cells occurs at lower rates in the absence of spleen. In consequence, splenectomized BLV-infected sheep accumulate B cells with extended lifetime. This process may favor accumulation of genetic defects leading to a higher probability of transformation and reducing latency period before onset of leukemia.

We have demonstrated here that the spleen is a major component of the host immune control of viral pathogenesis. Cell kinetics measurements show that B cells in splenectomized sheep have a defect in cell death compared to controls (Figure 2). It is likely that this disequilibrium accelerates onset of leukemia in splenectomized animals. However, CFSE labeling data show that an excess of about 6.4% of peripheral B cells would accumulate per day, providing that all other parameters remain constant. Since very fast onset of leukemia was definitely not observed immediately after surgery (i.e. within days), other regulatory mechanisms are predicted to maintain homeostasis such as enhanced recirculation to other lymphoid organs, decreased cell production/differentiation or accumulation in other tissues.

The mechanism outlined in this report may also be informative for the pathogenesis induced by the related HTLV-1 in particular for ATL. Although splenectomy has recently been proposed as an optional treatment for hypersplenism caused by ATL [24], the consensus policy includes chemotherapy, antiviral therapy and allogeneic hematopoietic stem-cell transplantation [25]. In specific cases, splenectomy may therefore be considered as a palliative treatment for pain relief. The observations of this report enlighten another important aspect of asplenism in HTLV carriers. When splenectomy has to be performed due to traumatic or possibly diagnostic purposes, there is a potential risk for accelerating onset of ATL in these subjects. Therefore, HTLV-1 infected asymptomatic carriers undergoing splenectomy would require a more regular preventive evaluation of their clinical stage.
